# A Portable Low-Power Acquisition System with a Urease Bioelectrochemical Sensor for Potentiometric Detection of Urea Concentrations

**DOI:** 10.3390/s16040474

**Published:** 2016-04-02

**Authors:** Wei-Jhe Ma, Ching-Hsing Luo, Jiun-Ling Lin, Sin-Houng Chou, Ping-Hung Chen, Mei-Jywan Syu, Shin-Hung Kuo, Shin-Chi Lai

**Affiliations:** 1 Instrument System and Chip Group, Department of Electric Engineering, National Cheng Kung University, Tainan 70101, Taiwan; harukokashi@gmail.com (W.-J.M.); guosuicune@hotmail.com (S.-H.K.); 2 Institute of Medical Science and Technology, National Sun Yat-sen University, Kaohsiung 80424, Taiwan; 3 Biotechnology and Biochemical Engineering Group, Department of Chemical Engineering, National Cheng Kung University, Tainan 70101, Taiwan; 4 Department of Computer Science and Information Engineering, Nan Hua University, Chiayi 62249, Taiwan; shivan0111@nhu.edu.tw

**Keywords:** portable, battery-driven, bioelectrochemical signal acquisition, urea, potentiometric, immobilized urease, MEMS

## Abstract

This paper presents a portable low-power battery-driven bioelectrochemical signal acquisition system for urea detection. The proposed design has several advantages, including high performance, low cost, low-power consumption, and high portability. A LT1789-1 low-supply-voltage instrumentation amplifier (IA) was used to measure and amplify the open-circuit potential (OCP) between the working and reference electrodes. An MSP430 micro-controller was programmed to process and transduce the signals to the custom-developed software by ZigBee RF module in wireless mode and UART in able mode. The immobilized urease sensor was prepared by embedding urease into the polymer (aniline-co-*o*-phenylenediamine) polymeric matrix and then coating/depositing it onto a MEMS-fabricated Au working electrode. The linear correlation established between the urea concentration and the potentiometric change is in the urea concentrations range of 3.16 × 10^−4^ to 3.16 × 10^−2^ M with a sensitivity of 31.12 mV/log [M] and a precision of 0.995 (R^2^ = 0.995). This portable device not only detects urea concentrations, but can also operate continuously with a 3.7 V rechargeab-le lithium-ion battery (500 mA·h) for at least four days. Accordingly, its use is feasible and even promising for home-care applications.

## 1. Introduction

The urea concentration in human blood, called blood urea nitrogen (BUN), is significant for the assessment of normal renal function. BUN, a metabolic waste product of protein, is secreted by the kidneys and excreted from the body via urine. The BUN of a healthy adult ranges between 6 and 21 mg/dL (1–3.5 mM). Renal failure and related complications, such as acute or chronic urinary tract obstruction, hepatic failure and nephritic syndrome, could causes in urea concentration [1,2,3,4]. Currently, more than two thousand people per million worldwide rely on hemodialysis. Normally, hemodialysis is required when the urea concentration in serum is above 180 mg/dL. BUN is temporarily raised in some diseases or human conditions such as liver dysfunction, upper gastro-intestinal bleeding, hydropenia, or hepatitis. Thus, it is essential to develop a device that can measure urea concentration rapidly and precisely.

The urease catalytic reaction mechanism for urea is as follows:

(1) $$NH_{2}CONH_{2}+3H_{2}O+H^{+}\overset{urease}{\to }HCO_{3}^{-}+OH^{-}+2NH_{4}^{+}$$

According to Equation (1), the determination of urea concentration depends on the formation of ammonia or the change of pH [5]. In this manner, an electrochemical urea sensor can accurately measure the urea concentration.

A recent surge of research into bioelectrochemical sensors has concentrated on sensor fabrication via micro-electro-mechanical system (MEMS) technology. Many MEMS-based bioelectrochemical sensors for different applications have been described, such as *E. coli* bacteria detection [6], nitric oxide sensing [7], glucose detection [8], and DNA hybridization [9]. Each of these papers targeted the urea concentration via fabrication of a bioelectrochemical sensor by the MEMS technique.

In the past few decades, electrochemical sensors have been widely applied in various research fields owing to their high selectivity and sensitivity, rapid analysis, simplicity, easy fabrication and low cost [10,11]. These electrochemical sensors serve as transducers that convert a chemical concentration into an analog signal for measurement by an electronic device. Additionally, by physical entrapment and covalent bonding [12,13,14,15,16,17,18], urease has been immobilized onto a working electrode to achieve electropolymerization [19,20]. So far, all research efforts have been aimed at immobilizing urease within different kinds of polymeric matrices and with minimum leakage and loss of activity. To measure the urea concentration, various studies designed electrochemical urea sensors based on conductometry, potentiometry and amperometry with flow injection analysis (FIA). In recent years, much attention has been given to various urea biosensor designs, such as a solid-state urea biosensor [21] in the potentiometric mode, a gold interdigitated microelectrode urea biosensor based on polyhydroxybutyrate substrate in the impedimetric mode [22], and a carbon-black electrode urea sensor based on the amperometric mode [23,24,25]. Li *et al.* [26] developed a conducting polymer hydrogel amperometric biosensor for measuring human metabolites, such as uric acid, cholesterol and triglycerides, in a wide linear range. They reviewed the design and fabrication of such an electrode—Including different-dimensional nanostructures and biosensor applications [27,28]. More generally, these studies highlighted the importance of urea biosensors for bioelectrochemical sensing applications.

Recently, increasing attention has been devoted to research into personal health-care due to the globally aging population and chronic diseases. To meet the demands of personal health-care, the miniaturization of medical monitoring instruments into portable devices has become an important issue for health-care applications. Some commercial products based on the concept of point-of-care (home-care) service as well as long-term monitoring have already begun to appear on the market, such as portable and wearable blood glucose meters and continuous electrocardiogram (ECG) monitors [29,30,31,32,33,34]. In line with this trend, the main aim of the present study was to develop a rapid biomedical monitoring device.

**Figure 1:**
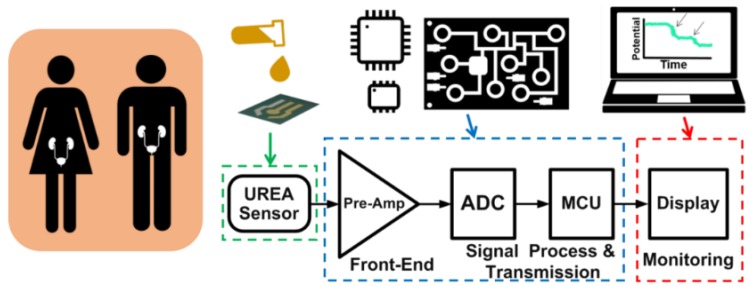
The proposed bioelectrochemical acquisition system prototype.

Over the past few decades, much research has focused on portable biomedical devices that offer simple and reliable biomedical detection. In this regard, bioelectrochemical acquisition systems can acquire signals quickly and accurately. Some studies have used discrete components and evaluation board platforms to realize bioelectrochemical acquisition applications [35,36,37,38,39], while other works have concentrated on application-specific integrated circuit (ASIC) chips in single-, dual-, or multi-type electrochemical biosensor-integrated device studies [40,41,42,43]. In the present study, a bioelectrochemical acquisition system prototype device was realized as a biosensor for the kidney function. More specifically, the present paper proposes a bioelectrochemical monitoring prototype for the analysis of renal function by targeting urea concentration both in rapid detection and long-term monitoring. In this study, to increase the autonomy of users and improve the quality of detection, we focused on speed, convenience, performance and low-power consumption. Figure 1 schematically depicts our proposed bioelectrochemical acquisition prototype device for rapid analysis of renal function. As shown, it comprises a MEMS-based urea sensor, a front-end circuit, a signal-processing unit, an RS-232 driver, a ZigBee module, and custom-developed software for the monitoring. The proposed urea bioelectrochemical sensor based on a silicon substrate is fabricated by a MEMS technique and responds in the potentiometric mode. The proposed front-end circuit is linked to a 12-bit ADC, which converts the analog signal into a digital sequence. Furthermore, the microcontroller unit processes and transmits the data to a computer or any receiving device by two modes, namely a ZigBee RF module in wireless mode and a UART in cable mode. Finally, the custom-developed software features a user-friendly interface for data display and storage.

To sense urea, the bioelectrochemical module is composed of three electrodes: A Au working electrode, a Au counter electrode, and a Ag/AgCl reference electrode. The gold and silver-based electrodes are manufactured by a MEMS process. The silver-based reference electrode is oxidized by an after-process treatment to form a Ag/AgCl electrode, after which the immobilization of urease is performed by physical entrapment using electropolymerization.

The experimental results revealed that the system was able to achieve excellent performance in the calibration of urea concentration against potentiometric change. Accordingly, it would be suitable for application in rapid analysis and monitoring of renal function, for instance, in future development of a low-power wireless biotelemetry system-on-a-chip (SoC) device.

## 2. Materials and Methods

### 2.1. Fabrication of the MEMS-Based Bioelectrochemical Sensor

The proposed design of the electrochemical sensor for urea detection involved a three-electrode system fabricated by a MEMS technique. The electrochemical module consisted of three electrodes on a silicon substrate as the urea sensing area, comprising a Au working electrode, a Au counter electrode and a Ag/AgCl reference electrode. The sensor’s working electrode is the one that plays the principal role in urea sensing. Next, the counter electrode of the sensor was constructed from another gold electrode, while the reference electrode was a pseudo (quasi) Ag/AgCl electrode [44,45]. The gold on the electrodes was deposited via e-beam evaporation, with the metallization utilized for interconnecting the metal traces and testing pads.

The fabrication process involved three steps: Standard photolithography, thin-film deposition and lift-off. A 4'' phosphorus-doped silicon wafer (100) with a thickness of 500 µm was used as the silicon substrate (Ultimate Materials Technology Co., Pingtung, Taiwan). The substrate was first cleaned using the standard RCA cleaning steps. Afterwards, a low-stress 1.0 µm-thick Si_3_N_4_ layer was deposited on the silicon substrate as the isolation layer on each side via a low-pressure chemical vapor deposition system (LPCVD) (NFC-NCTU, Hsinchu, Taiwan). The operation conditions to deposit the low-stress Si_3_N_4_ thin film were: SiH_2_Cl_2_ (85 sccm), NH_3_ (175 sccm), 50 mtorr pressure, and a temperature of 850 °C.

**Figure 2:**
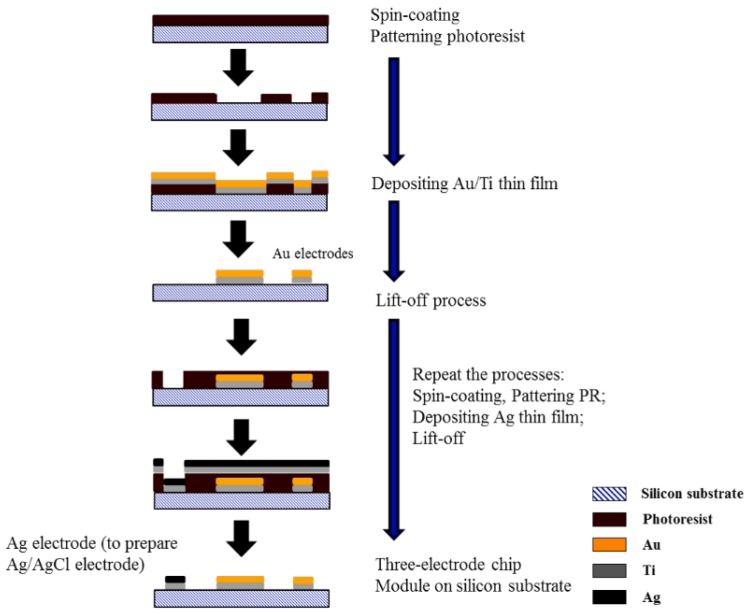
Three-electrode chip molding process flow.

The first step in fabricating the three-electrode layer from the MEMS process involved spin-coating the AZ1500 photoresist with a thickness of 1.5 ctrode layer from the MEMS proces_3_N_4_), and then setting the pattern with a Mask Aligner (MA 150CC, SUSS Micro-Tec, Garching, Germany). Then, a Ti (15 nm) layer was deposited to enhance the adhesion of Au to the silicon surface, after which e-beam evaporation was used to deposit the Au layer (120 nm). The pattern on the Au layer was then created by a standard lift-off process. In this manner, both the Au electrodes namely the working and counter electrodes were fabricated. Also the fabrication of the Ag electrode (120 nm) followed the above-mentioned process. The metal-deposition conditions are summarized in Table 1, with each layer deposited in two steps. The metal-deposition rate of the first step was 10 times slower than that of the second step. However, the two-step metal-deposition procedure was used because it gave superior adhesion and film quality. After finishing the micro-fabrication of the three electrodes, the Ag electrode was oxidized in a FeCl_3_ (100 mM) solution to produce the Ag/AgCl reference electrode. Finally, the fabrication process was completed by washing the three electrodes in distilled water. A flow chart of the entire process is shown in Figure 2.

### 2.2. Electrochemical Detection of Urea Concentration

The potentiometric mode was used to detect the urea concentration. Three electrodes, namely the working, counter and reference electrodes, were needed to perform the electropolymerization. The conductive materials not only enhanced the conductivity of the electrodes, but also trapped the urease enzyme which detected the urea. Cyclic voltammetry was used to trigger the electropolymerization for the formation of the conductive polymeric matrix on the Au working electrode.

By physical entrapment, urease (E.C. 3.5.1.5, from Jack Beans, Type III, Sigma-Aldrich Inc., St. Louis, MO, USA, crude enzyme without further purification or pretreatment before use) in a concentration of 5.0 mg/mL was immobilized within the conductive polymeric matrix. Furthermore, urease was mixed with aniline (ANI) monomer (Sigma-Aldrich Chemie GmbH, Buchs, Switzerland) and *o*-phenylenediamine (*o*-PD; Sigma-Aldrich Chemie GmbH), after which cyclic voltammetric polymerization was initialized for the synthesis of poly(ANI-co-*o*-PD), the polymer, poly(aniline-co-*o*-phenylenediamine) as well as for the immobilization of urease. Polyaniline is a well-known electroconducting polymer in a linear chain. However, it is difficult to firmly entrap urease only by polyaniline. Therefore, *o*-phenylenediamine was used to add a branching structure into the polymer matrix for enhancing the immobilization of urease. The scan range was from −0.2 to 1.0 V, with a scan rate of 50 mV/s over 10 cycles (*i.e.*, 20 segments). To detect the urea concentration, urease catalyzed the reaction of urea into ammonia, as shown in formula (1), so that a corresponding potential-change signal was obtained. Instead of the three-electrode system, only the working and reference electrodes (a two-electrode process) were needed to detect the urea concentration. The open-circuit potential (OCP) between the working and reference electrodes was measured and correlation of the urea concentration was established based on the Nernst equation.

### 2.3. Low-Power Battery-Driven Bioelectrochemical Acquisition System

**Figure 3:**
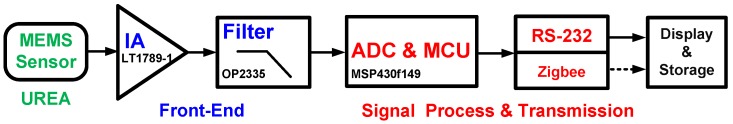
Block diagram of the proposed bio-electrochemical acquisition system.

The block diagram of the battery-driven portable potentiometric device designed in this work is shown in Figure 3. The open-circuit potential (OCP) obtained from the sensing electrode (*i.e.*, the polymer immobilized urea on the MEMS-base substrate) was amplified and filtered by the front-end circuit, which included an instrumentation amplifier and a low-pass filter. The low-pass filter was composed of an operation amplifier and passive components. Next, the analog signals were converted and processed by a commercial MCU, which included a 12-bit ADC. Afterwards, the digital signals were transmitted to a laptop computer by RS-232 serial communication protocol (MAX3232E-Q1, Texas Instruments Inc., Dallas, TX, USA) in cable mode, or an IEEE 802.15.4/ZigBee-compliant transceiver in wireless mode. Finally, the data were further plotted and stored by the custom-developed software.

### 2.4. Bioelectrochemical Readout Circuit

Herein, the urea detection was mainly aimed at potential responses between the electrochemical cell working electrode (WE) and the reference electrode (RE). The potential response of the proposed urea sensor is called the open-circuit potential, which involves no current flow through the WE and RE during the experiments; accordingly, the input impedance of the terminals connected to the WE and RE must be as high as possible. In addition, the electrochemical cell WE and RE of the urea sensor were linked to the proposed bioelectrochemical readout circuit for the bioelectrochemical signal acquisition.

**Figure 4:**
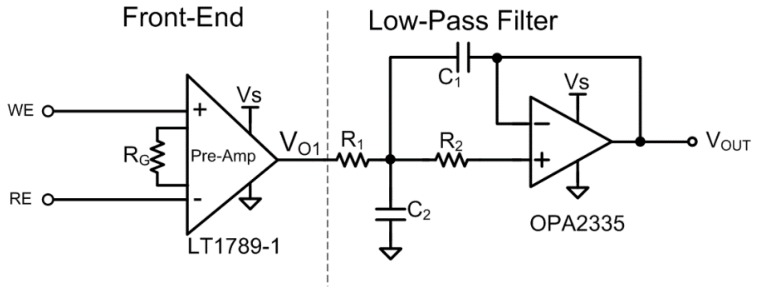
The proposed bio-electrochemical readout circuit for OCP measurement.

Figure 4 schematically depicts the proposed readout circuit, which includes two parts: The front-end amplifier and low-pass filter. The front-end amplifier of the readout circuit must possess the following characteristics: a high common mode rejection ratio (CMRR), high input impedance, low-power consumption, and low noise for processing the bioelectrochemical signals. Therefore, a high-performance instrumentation amplifier (LT1789-1, Linear Technology, Milpitas, CA, USA) was adopted as the front-end readout circuit of the system. The utilized amplifier had a CMRR of 100 dB and a power supply rejection ratio (PSRR) of 100 dB. The WE and RE were connected to the differential inputs of the instrumentation amplifier, with R_G_ used to tune the gain for the readout potential change of the urea concentration. Accordingly, the gain of LT1789-1 can be represented as:

(2) $$Gain=\left[1+(\frac{200k\Omega }{R_{G}})\right]$$

Following the instrumentation amplifier circuit, a low-pass filter with a low cut-off frequency was selected to suppress the out-of-band noise and to provide better a signal-to-noise ratio (SNR). In addition, the low-pass filter was designed with an operational amplifier (OPA2335, Texas Instruments Inc., Dallas, TX, USA) to reduce the high frequency noise.

The gain of this instrumentation amplifier has the advantage that it can be tuned for various bioelectrochemical experiments by trimming the resistor value and the bandwidth of the filters can be designed to suit the system. The output of the readout circuit was connected to the input of the MSP430f149 ADC (Texas Instruments Inc., Dallas, TX, USA) for data processing and the UART unit for data transmission.

The LT1789-1 features low-power consumption, a high CMRR, high PSRR, high accuracy, rail-to-rail input and output range, and a wide supply voltage (2.2–36 V). Considering the purpose of the portable low-power system, the instrumentation amplifier (LT1789-1) was designed for low-voltage supply operation with a quiescent current of 95 μA.

### 2.5. Microcontrol Unit Usage

The proposed peripheral devices included an ADC and an MCU. Therefore, an ultra-low-power high-performance 16-bit RISC microcontroller (MSP430f149, Texas Instruments Inc., Dallas, TX, USA) with a 12-bit built-in ADC was selected for the system [46]. This integrates 2 KB of SRAM, and 48 KB + 256 bytes of flash memory for code programing. As mentioned above, the microcontroller featured a powerful 16-bit RISC CPU, 16-bit registers and constant generators that allow maximum code efficiency. Since the resolution of the built-in ADC is 12 bits, it is suitable for biomedical instruments and biophysical measurements.

The bioelectrochemical analog signals extracted by the analog front-end circuit are converted into digital data by the built-in ADC. Next, the MCU processes the digital signals and sends them to the computer for data display and storage via the UART or radio frequency module. The personal computer then acquires the signals through the peripheral devices and processes them using the custom-develop software.

The architecture of the commercial low-power MSP430f149 MCU consists of two built-in 16-bit timers, a 12-bit ADC, two UARTs, 48 I/O pins and small size and weight. In addition, it has one active mode and five software selectable power-saving modes; thus, it is optimized to achieve an extended battery life for portable measurement applications. In power-saving mode, the digitally controlled oscillator (DSC) can wake up from a low-power mode to active mode in less than 6 µs, including performing the functions of power-management control, enabling instrumentation amplifier, ADC and processing the signals. Furthermore, under the active mode, the active current is 280 µA at 1 MHz, while the idle current is 1.6 µA in standby mode.

### 2.6. Peripheral Device for Signal Monitoring and Storage

The UART interface is widely used for serial communication and its transport protocol is simpler than that of the universal serial bus (USB). Although UART has relatively slow data transmission, it is sufficient for this work due to the long response time of the sensors.

The peripheral devices of the proposed system transmit the acquired bioelectrochemical signal to the computer for data display and storage. In front of the peripheral device, the MCU processes and sends the signals via a UART or wireless transmission solution. Furthermore, a personal computer or laptop computer acquires the signals through the peripheral devices and displays them by using system designed software. The present study employed two modes of data transmission for the proposed data acquisition, as described below.

In cable mode, the selected MSP430f149 MCU has two UART interfaces; thus, the proposed portable device can be connected to a computer via a USB, a UART port and/or a RS-232 serial protocol. A multi-channel RS-232 line driver/receiver (MAX3232E-Q1, Texas Instruments Inc. Texas Instruments Inc., Dallas, TX, USA) was used to meet the requirements of TIA/EIA-232-F (RS-232) and to provide the electrical interface between the asynchronous communication controller and the serial-port connector.

In wireless mode, the chosen wireless module was an IEEE 802.15.4/ZigBee-compliant transceiver. It contains a powerful 8-bit 8051 MCU and an RF single-chip (CC2530, Texas Instruments Inc., Dallas, TX, USA) 2.4 GHz IEEE 802.15.4-compliant RF transceiver. The RF transceiver module is employed as a communication transceiver in bidirectional telemetric devices. Accordingly, a wireless sensor network can be built from the transceiver modules for various applications. The transmission range of the transceiver is 100 m (LOS). Additionally, the receiver unit receives UART formatted data transmitted via a USB port on a UART/USB interface IC (FT232RL, FTDI Chip Inc., Glasgow, Scotland).

The proposed custom-developed software was written in Visual Basic and Lab-View to provide a user-friendly interface to display the data in real-time and to automatically upload the data to a database via the Internet.

## 3. Results and Discussions

### 3.1. Urea Disposable Sensing Microchip

**Figure 5:**
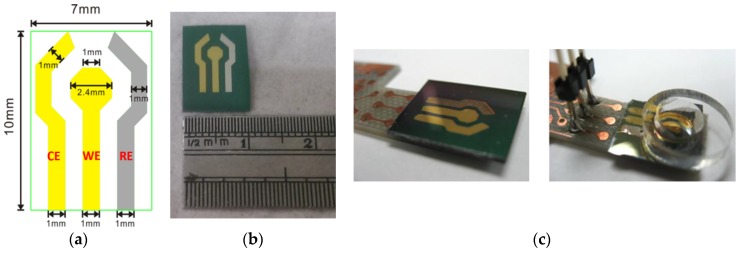
(**a**) Schematic diagram of the proposed urea sensor chip; (**b**) Photo of the urea sensor; (**c**) Photos of the disposable urea sensor integrated with the PCB (**Left**) and PDMS (**Right**).

A three-electrode system functioning as a urea sensor, as well as a system for detecting urea concentration, was fabricated by a MEMS technique. A schematic diagram of the fabricated three microelectrode urea sensor prototype is shown in Figure 5a. As can be seen, the diameter of the sensing area is 2.4 mm, while those of the counter and reference electrodes are both 1.0 mm. The total area of the urea sensor is 70 mm^2^, with an actual sensing area of 4.78 mm^2^ on the working electrode. Additionally, polydimethylsiloxane (PDMS) was used to create a reservoir for the urea samples. Detection of the urea concentration was carried out once the urease enzyme was immobilized on the Au working electrode via the formation of a film of copolymer poly(ANI-co-*o*-PD), *i.e.*, poly(aniline-*co*-*o*-phenylenediamine) (*i.e.*, A photograph of the fabricated three-electrode electropolymerization urea sensing chip is presented in Figure 5b, with the proposed sensor integrated with the IC heterogeneously. Therefore, the proposed sensor was attached to a printed circuit board (PCB) and the reservoir channel holding the working electrode was made by PDMS, as shown in Figure 5c.

### 3.2. SEM Images of the Poly Film Fabricated on the Au Electrode

**Figure 6:**
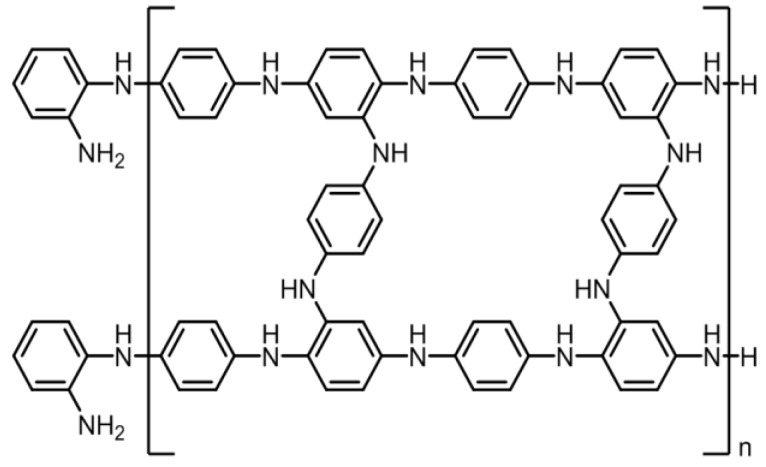
The chemical structure of poly(aniline-co-*o*-phenylenediamine).

**Figure 7:**
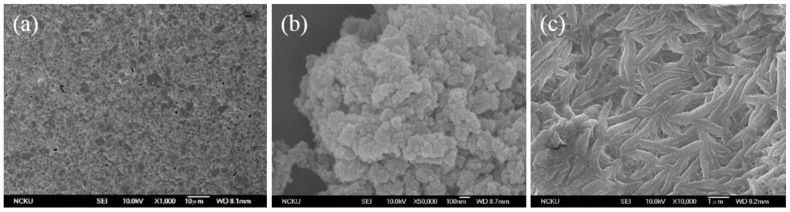
SEM images of the sensing surface of (**a**) Au; (**b**) polyaniline electro-fabricated onto Au; and (**c**) poly (aniline-co-*o*-phenylenediamine) on Au.

The chemical structure of poly(ANI-co-*o*-PD) is shown in Figure 6. Polyaniline can only provide electro-conductivity; however, it has a linear chain structure. Therefore, we used *o*-phenylenediamine to graft with aniline so that the copolymer thus fabricated could more firmly entrap urease. Figure 7a–c present the SEM images of the Au surface, the morphology of polyaniline and the morphology of the poly(ANI-co-*o*-PD) adhered onto Au, respectively. Obviously, the presence of *o*-phenylenediamine together with aniline, after electropolymerization, formed a kind of network structure, which could be seen on the surface of the copolymer film thus created, which then enhanced the immobilization effect of urease onto the electrode.

### 3.3. The Proposed Bioelectrochemical Signal Acquisition System

**Figure 8:**
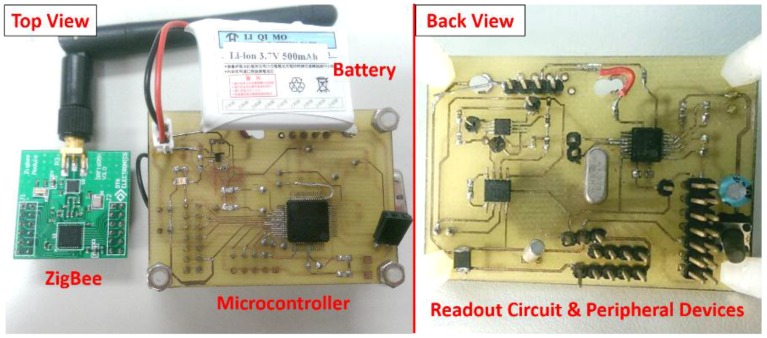
Realization of the proposed system device.

Portable biomedical instruments for the purpose of home-care application are becoming increasingly important. The proposed acquisition system presented in this paper was implemented and integrated with a MEMS urea sensor, a low-power bioelectrochemical readout circuit, an ultra-low-power MCU, a low-power RS-232 driver/receiver, and custom-developed software, with the entire system shown in Figure 8. The size of the proposed system device is 6.0 × 4.3 cm^2^ and the height is approximately 2 cm.

The proposed device and sensor for urea concentration detection are described in the following. First, the working and reference electrodes of the urea sensor were connected to the dual inputs of the readout circuit, and the open-circuit potential between them was measured; then, the potential signal from the sensor was amplified and linked to the ADC for signal conversion. The gain was set to 40 dB for this active component. The chosen MCU was programmed to initialize the built-in ADC, clock system and UART. The reference voltage provided for the built-in ADC and sampling timer was 3.3 V. In addition, the sampling timer (SHT_0) and the sampling time (*t*_ sample_ = 4·*t*_ADC12CLK_ ·8) were chosen for signal processing. The MCU clock was produced by an external 4 MHz crystal oscillator. The baud rate of the UART was 9600 bps and in cable mode, the peripheral device included an MCU and UART/RS232 communication chip (MAX3232E-Q1). The MCU obtains biomedical analog signals by using the proposed processing chip and transmits the signals via UART to the PC. Furthermore, in wireless mode, the device contains a MCU and wireless ZigBee module (CC2530); accordingly, the MCU can transmit biomedical signals wirelessly.

The factors of long-term monitoring, low-power consumption, low cost, and compact size must be taken into consideration. In cable mode, the total power consumption of the proposed design is 12.42 mW with a supply voltage of 3.3 V. Consequently, the power dissipation of the proposed system device is considered a low-power design and therefore suitable for battery-driven operation and long-term monitoring. The MCU can transmit the biomedical signals wirelessly with a power dissipation of 32.62 mW. A wireless solution is more appropriate for portable monitoring applications, despite consuming more power. Moreover, this system is very stable and can be continuously operated for at least 4 days with a 500 mA·h battery in cable mode; by contrast, the device lifetime in wireless mode is about 2 days. Table 2 summarizes the power consumption of the proposed readout system device in dual mode.

The proposed battery-driven device is powered by a 3.3 V regulated external power supply. Therefore, the power supply of this device consisted of a +3.3 V voltage regulator commercial IC, TPS76933 and bulk decoupling capacitors. The TPS769 family is a low-dropout-voltage and low-noise regulator; accordingly, it is suitable for portable applications. The TPS769 also features a logic-enabled sleep mode to shut down the regulator, thereby reducing quiescent current to 1 µA, which makes the IC appropriate for use in battery-powered devices. A rechargeable 3.7 V Li-ion 500 mA·h battery pack provided the power to the proposed battery-driven device.

### 3.4. Detection of Urea Concentration by the Proposed System Device and Sensor

**Figure 9:**
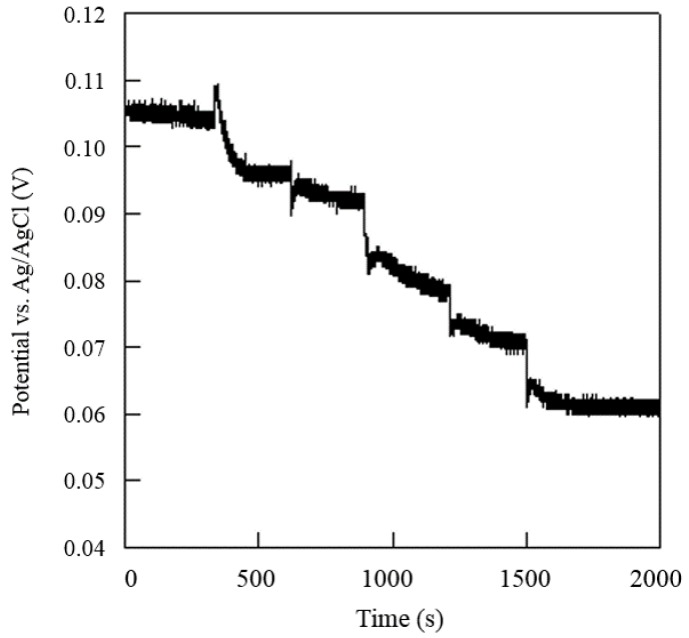
Potential time profile in response to the injection of urea solution.

**Figure 10:**
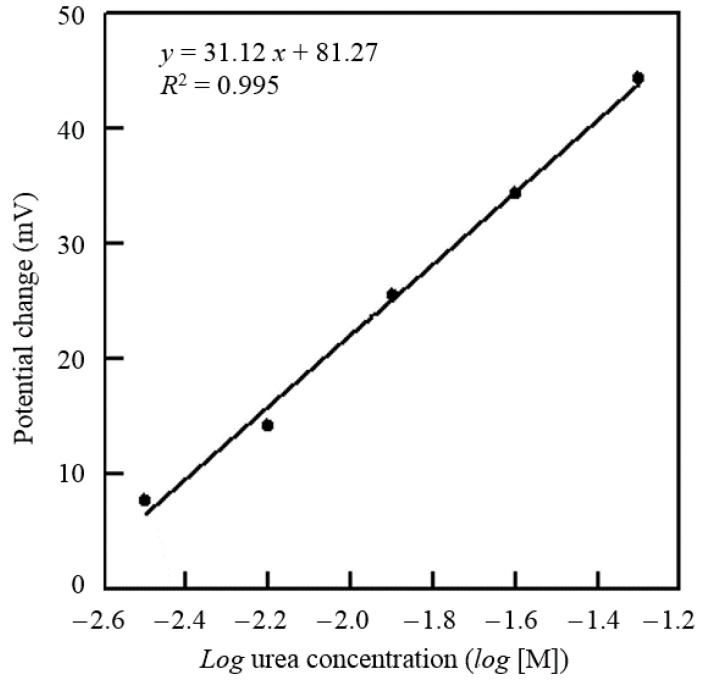
Characteristic curve of urea concentration in test samples.

In this paper, electrochemical analysis experiments were conducted with the proposed system device and urea electrochemical sensor. The urea concentration measurement was performed by the sequential injection of urea up to a working volume of 50 μL. The urea concentration of the proposed system device was calibrated by using a commercial potentiostat (CHI-611C, CH Instruments Inc., Tennison Hill Drive Austin, TX, USA) as the standard. Figure 9 shows the time profile of the corresponding voltage changes in response to the sequential injections of urea. Figure 10 shows the potential variations against the urea concentrations obtained. The minimum urea concentration required that could be detected was experimentally found to be 3.16 × 10^−4^ M (1.89 mg/dL) with the pH set at 7.4. Furthermore, a sensitivity of 31.12 mV/log [M] could be achieved in the urea concentration range of 3.16 × 10^−4^ M to 3.16 × 10^−2^ M (1.89–189.93 mg/dL) with a precision of 0.995 (R^2^ = 0.995). With a sample volume of only 10 μL, accurate measurement of the urea concentration could be obtained in less than 100 s.

According to the Nernst equation, the sensing results can be reasonably explained. Moreover, the results confirm the successful measurement of urea concentrations by this portable device. We further compared the sensing results in this work with our previous work [47]. The major differences are listed as follows. First, the proposed battery-driven portable device in the present study replaces the function of a potentiostat, while in our previous work the sensing system was operated by a potentiostat. Second, the MEMS in our previous work was designed with a microchannel for the pumping of microfluid to the sensing area, while in the present work the MEMS has no pumping action. A comparison of the sensing performance for the present work and our previous work is listed in Table 3. With an injection volume of 10 μL to a working volume of 50 μL whole serum, the linear calibration had a sensitivity of 1.59 ± 0.47 mV/log [M] for serum urea concentration, which was similar to [47]. The experimental results of the integrated device and sensor are summarized in Table 4, while Table 5 compares the differences between the system proposed in the present work and the design described by Wang *et al.* [40].

## 4. Conclusions

To achieve the requirements of low-power consumption, high portability, and low cost, we proposed a low-power battery-driven portable device in this paper. The performance of this device was successfully confirmed via its integration with a disposable urea sensor. The proposed system device includes the following modules: A low-supply-voltage IA, 2nd-order low-pass filter, low-power microcontroller, a RS-232 driver/receiver, an IEEE 802.15.4/ZigBee-compliant transceiver and custom-developed software. The potential response results of the urea sensor show that the sensitivity is 31.12 mV/log [M], which was achieved in the urea concentration range of 3.16 × 10^−4^ M to 3.16 × 10^−2^ M (1.89–189.93 mg/dL). This low-power portable device can not only quantify the urea concentration, but also stably measure urea signals for at least four days running on a 3.7-V Li-ion battery. Consequently, this low-power portable device holds potential and its use in biosensors for home health care applications is feasible.

## Figures and Tables

**Table 1:** Deposition conditions of metal layers (Ti, Au and Ag) on the substrate.

	Ti	Au	Ag
1st Deposition rate	0.01 nm/s	0.01 nm/s	0.01 nm/s
1st Thickness	5 nm	5 nm	5 nm
2nd Deposition rate	0.1 nm/s	0.1 nm/s	0.1 nm/s
2nd Thickness	10 nm	115 nm	115 nm
Vacuum	3 × 10^−6^ torr		

**Table 2:** Power consumption of the proposed readout system device in dual mode.

Cable Mode	Front-End	MCU	MAX3232	Regulator
P.C. (mW)	0.32	7.88	3.31	0.92
% of Total P.C.	2.57%	63.40%	26.63%	7.40%
Total Power	12.43 mW			
Device lifetime	4–5 days (A 3.7V Li-ion 500 mA·h battery)			
Wireless mode	Front-end	MCU	ZigBee	Regulator
P.C. (mW)	0.32	7.88	23.5	0.92
% of Total P.C.	0.98	24.15%	72.04%	2.82%
Total Power	32.62 mW			
Device lifetime	1–2 days (A 3.7V Li-ion 500 mA·h battery)			

**Table 3:** Comparison between the proposed sensor and previous work.

Specification	[47]		This Work	
Background Solvent	water	serum	water	serum
Sensitivity (mV/log [M])	28.68 ± 0.01	2.71 ± 0.56	31.12	1.59 ± 0.47
Sampling Time (s)	400	400	100	100
Response Time (s)	~800	~1200	~100	~500
Sample Volume (mL)	0.20	0.20	0.01	0.01
Working Volume (mL)	5	5	0.05	0.05

**Table 4:** Specifications results of the proposed system and sensor.

Key Item	Specification	
System Power Supply	3.3 V	
Power Consumption	Cable	ZigBee
(Cable mode & Wireless mode)	12.42 mW	32.62 mW
Linearity (R^2^)	0.995	
Sensitivity (mV/log [M])	31.12	
UART Baud rate	9600	
Device Size	6.0 × 4.3 cm^2^	
Sensor Size	0.7 × 1.0 cm^2^	

**Table 5:** Comparison between our system and previous work.

Specification	[40]	This Work
Circuit Composition	CMOS/FPGA	Commercial Chip
Power Supply	1.8V/3.3 V	3.3 V
Linearity	0.998	0.995
Power consumption	157.25 mW	32.62 mW
Data transmission	433 MHz/ISM	2.4 GHz/ZigBee
